# Supplementary material for: Comparing Plasma Exchange to Escalated Methyl Prednisolone in Refractory Multiple Sclerosis Relapses
Source: J Clin Med. 2019 Dec 22;9(1):35. doi: 10.3390/jcm9010035 (PMC7027010; doi:10.3390/jcm9010035)
Supplement: Supplementary file 1 [file jcm-09-00035-s001.pdf]

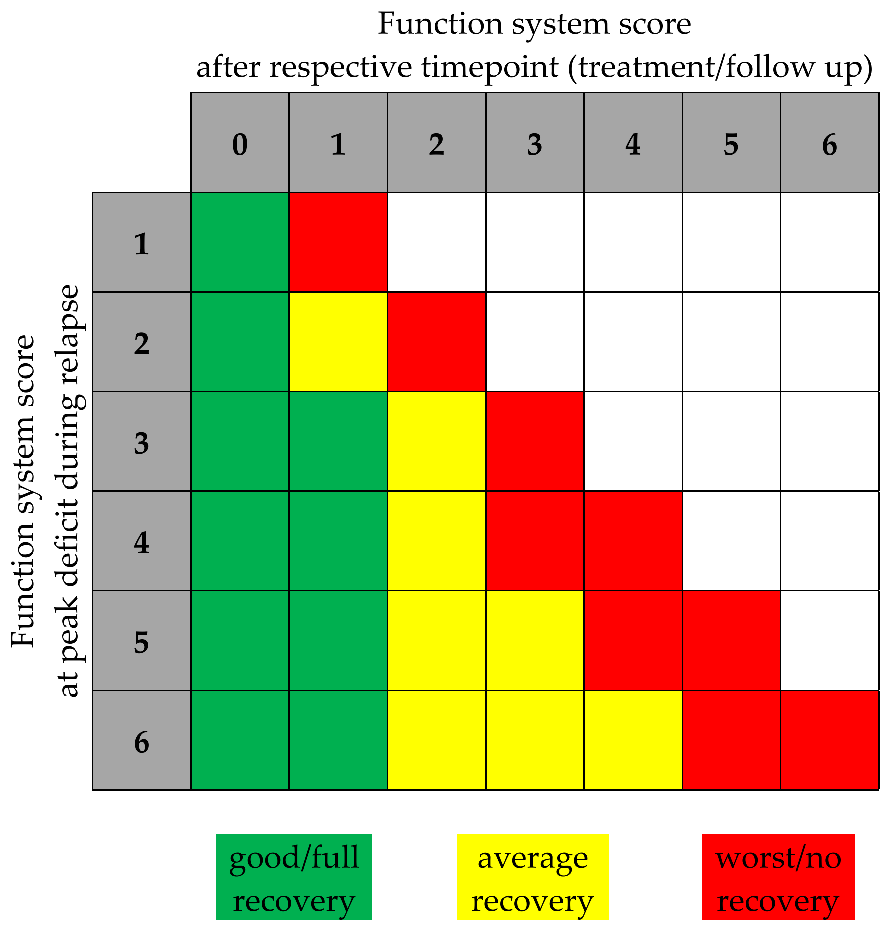

Figure S1: Functional systems score (FSS) based relapse recovery model.

Matrix showing stratification of treatment recovery according to function system scores (FSS) at peak deficit and after treatment. In our study, we accepted recovery to baseline FSS as "good recovery" irrespective of this matrix. The matrix was modified from the original variant proposed by Conway and colleagues [13].

	TPE (n=46)	IVMPS (n=99)	total	p-value
**treatment-naïve**	*30 (65.2)*	*60 (60.6)*	*90*	*0.694*
**beta-interferon[1]**	*5 (10.8)*	*9 (9.9)*	*14*	
**glatiramer acetate[1]**	*0 (0)*	*8 (8.08)*	*8*	
**dimethyl fumarate[1]**	*3 (6.5)*	*5 (5.05)*	*8*	
**teriflunomide[1]**	*1 (2.2)*	*2 (2.02)*	*3*	
**fingolimod[2]**	*1 (2.2)*	*4 (4.04)*	*5*	
**natalizumab[2]**	*4 (8.7)*	*6 (6.06)*	*10*	
**alemtuzumab[2]**	*2 (4.3)*	*4 (4.04)*	*6*	
**daclizumab[2]**	*0 (0)*	*1 (1.01)*	*1*	

Table S1: Description of administered disease-modifying treatment at relapse onset. Total patient numbers are shown. P-value was determined using Fisher's exact test. [1]: Substances included in DMT group "approved for mild to moderate RRMS", [2]: Substances included in DMT group "approved for active RRMS".

	Odds ratio	95%-confidence interval	p-value
**IVMPS vs. TPE (ref.)**	*39.008*	*10.423-142.710*	***<0.001***
**Age**	*0.998*	*0.955-1.044*	*0.942*
**Sex (male vs. female (ref.))**	*1.144*	*0.446-2.938*	*0.779*
**Affected function system (visual vs. others (ref.))**	*0.939*	*0.391-2.255*	*0.939*
**Disease duration since onset**	*1.001*	*0.833-1.203*	*0.784*
**Baseline EDSS**	*0.784*	*0.450-1.365*	*0.390*
**Time to treatment initiation**	*1.028*	*0.908-1.164*	*0.666*
**Ongoing disease-modifying treatment (yes vs. no (ref.))**	*0.561*	*0.185-1.698*	*0.306*

Table S2: Results table from multivariable regression analysis with "worst or no treatment response following first escalation treatment" as dependent variable.

	Odds ratio	95%-confidence interval	p-value
**no TPE vs. TPE (ref.)**	*6.651*	*2.522-17.540*	***<0.001***
**Age**	*0.978*	*0.928-1.031*	*0.408*
**Sex (male vs. female (ref.))**	*1.771*	*0.568-5.524*	*0.325*
**Affected function system (visual vs. others (ref.))**	*0.415*	*0.146-1.185*	*0.101*
**Disease duration since onset**	*0.999*	*0.816-1.223*	*0.994*
**Baseline EDSS**	*1.184*	*0.670-2.093*	*0.562*
**Time to treatment initiation**	*0.932*	*0.807-1.076*	*0.334*
**Ongoing disease-modifying treatment (yes vs. no (ref.))**	*0.530*	*0.154-1.819*	*0.313*

Table S3: Results table from multivariable regression analysis with "stable course vs. further deterioration at follow-up" as dependent variable.

covariate	Odds ratio	95%-confidence interval	p-value
TPE as second vs. TPE as first escalation (ref.)	*4.628*	*1.346-15.913*	***0.015***
Age	*0.970*	*0.911-1.032*	*0.328*
Sex (male vs. female (ref.))	*0.391*	*0.106-1.441*	*0.158*
Affected function system (visual vs. others (ref.))	*1.553*	*0.452-5.345*	*0.485*
Disease duration since onset	*0.619*	*0.421-0.910*	***0.015***
Baseline EDSS	*1.975*	*0.969-4.023*	*0.061*
Time to treatment initiation	*0.526*	*0.384-0.721*	***0.000***
Ongoing disease-modifying treatment (yes vs. no (ref.))	*1.723*	*0.378-7.863*	*0.482*

Table S4: Results table from multivariable regression analysis with "development of severe adverse events" as dependent variable.
